# Fyn Tyrosine Kinase Increases Apolipoprotein E Receptor 2 Levels and Phosphorylation

**DOI:** 10.1371/journal.pone.0110845

**Published:** 2014-10-23

**Authors:** Teal C. Burrell, Shailaja D. Divekar, Edwin J. Weeber, G. William Rebeck

**Affiliations:** 1 Department of Neuroscience, Georgetown University Medical Center, Washington, District of Columbia, United States of America; 2 Department of Molecular Pharmacology and Physiology, University of South Florida Health Byrd Alzheimer’s Institute, University of South Florida, Tampa, Florida, United States of America; INSERM U901, France

## Abstract

Apolipoprotein E Receptor 2 (ApoER2) and the tyrosine kinase Fyn are both members of the Reelin pathway, a signaling pathway essential for the laminar formation of the cortex during development and proper dendritic spine density and long-term potential (LTP) in the adult brain. In the presence of extracellular Reelin, ApoER2 binds the intracellular protein Dab1, an adaptor protein that is phosphorylated by Fyn. However, direct interactions between ApoER2 and Fyn are not well defined. Here, we show that total levels of ApoER2 and surface levels of ApoER2 are increased by active Fyn. Via a separate mechanism, ApoER2 is also phosphorylated by Fyn, an event that peaks in the postnatal cortex at day 5 and can occur at multiple ApoER2 tyrosine residues. Dab1 is also involved in this phosphorylation, promoting the phosphorylation of ApoER2 by Fyn when it is itself phosphorylated. These results elucidate some of the intracellular mechanisms that give rise to a functional Reelin pathway.

## Introduction

The Reelin pathway helps to establish the laminar organization of the cortex, hippocampus, and cerebellum during brain development, and has a role in dendritic spine density and long-term potentiation (LTP) in the adult brain [1]–[3]. Reelin binds extracellularly to Apolipoprotein E Receptor 2 (ApoER2) and Very Low Density Lipoprotein Receptor (VLDLR), leading to receptor clustering and the phosphorylation of the intracellular adaptor protein Dab1 by the Src family kinase (SFK) Fyn [4]–[7]. These two Reelin receptors, ApoER2 and VLDLR, share overlapping functions and can compensate to some degree for the loss of the other [8]. However, ApoER2 has a greater role in the migration of late-born cortical neurons, in hippocampal layering, and in LTP, while VLDLR has a greater role as a stop signal to prevent over-migration in the cortex and in cerebellar structure [3], [9].

ApoER2 is a type I transmembrane protein with a large extracellular tail (705 amino acids) and a shorter intracellular tail (115 amino acids) [10] (NCBI Accession #001080926.1). The intracellular tail contains an NPXY domain, a well-characterized motif that signals internalization of extracellular ligands [11]. The NPXY domain of ApoER2 interacts with the phosphotyrosine binding domain of Dab1 [12], forming an important signaling complex [13]. Additionally, an exon encoding an intracellular domain, exon 19, is alternatively spliced [14] and has a role in Reelin-enhanced LTP, perhaps through interactions with PSD95 [15]–[16]. ApoER2 has three intracellular tyrosines, including one in the NPXY domain, and one in exon 19.

Fyn is a non-receptor tyrosine kinase of the Src family kinases that is myristoylated and associated with the cell surface [17]. Double knockouts to both *Fyn* and *Src* are very similar to *Reelin* knockouts, but Fyn seems to be the more crucial kinase as *Fyn* knockouts exhibit an intermediate phenotype and *Src* knockouts appear normal [18]. Reelin activates Fyn and this activation is dependent on the presence of ApoER2 and/or VLDLR and Dab1 [6]. Activated Fyn phosphorylates Dab1 [6]–[7], triggering downstream signaling that leads to the phosphorylation of phosphoinositide-3-kinase (PI3 K) and Akt, and the inhibition of glycogen synthase kinase 3β (GSK3β) [19], phosphorylation of n-cofilin [20], and increased NMDA receptor activity [21]. Phosphorylated Dab1 also interacts with Crk and CrkL, promoting the phosphorylation of the Rap1 guanine nucleotide exchange factor C3G, and leading to somal translocation, the final step in neuronal migration [22]–[27]. Through these various pathways, the extracellular binding of Reelin leads to the activation of Fyn and affects neuronal migration and translocation, dendritic spine development, and LTP.

By binding Reelin extracellularly [28] and Dab1 intracellularly [12], ApoER2 transfers the signal across the membrane, leading to the activation of Fyn. However, the molecular mechanisms behind how these interactions transduce a neuronal signal have not been elucidated. Fyn may help stabilize the receptor at the surface, providing the stimulus for interactions with Dab1. Here we show that ApoER2 levels are increased in the presence of Fyn and that ApoER2 is phosphorylated by Fyn, both *in vitro* and *in vivo*. Fyn also increases the interaction between ApoER2 and Dab1, and the presence of phosphorylated Dab1 increases Fyn’s ability to phosphorylate ApoER2. Together these results clarify interactions between ApoER2 and Fyn and reveal a novel detail–ApoER2 phosphorylation–of the Reelin pathway.

## Materials and Methods

### Vector Construction

ApoER2-myc and mutant ApoER2 constructs were created using the QuikChange Lightning Site-Directed Mutagenesis kit (Stratagene), according to the manufacturer’s protocol. Double-tagged ApoER2 (HA-ApoER2-myc) was created by inserting an HA tag after the endogenous ApoER2 signal peptide of the ApoER2-myc construct. ApoER2 lacking the intracellular domain (ΔICD) was created by inserting a stop codon into HA-ApoER2-Myc after W730, leaving only three intracellular amino acids intact. Untagged ApoER2 containing exon 19 and ApoER2 lacking exon 19 were kind gifts from Dr. Joachim Herz (University of Texas Southwestern). Tyrosine mutants to both the double tagged ApoER2 and the untagged ApoER2 were created sequentially, with single point mutations that changed each tyrosine to a phenylalanine. Whenever possible, experiments were performed on both sets of constructs (tagged and untagged) and representative blots are shown for one set for simplicity. VLDLR-myc was used as previously described [29]. GFP-tagged wild-type Dab1 was used, and Dab1 mutant constructs were made by point mutations of this construct. Constitutively-active Fyn [30] was used unless indicated otherwise. Kinase-dead Fyn was made by point mutation of wild-type Fyn (K299 M). All constructs were confirmed by sequencing and expression was confirmed by Western blot.

### Antibodies

We used antibodies anti-ApoER2 (Abcam, ab108208), anti-Fyn (Upstate), anti-tubulin (Sigma), anti-β-actin (Sigma), 4G10 (Millipore), anti-GFP (Invitrogen), anti-myc (abcam), and anti-HA (Abcam). All antibodies were used at 1∶1000 dilutions, except for *in vivo* immunoprecipitation blots, for which anti-ApoER2 was used at 1∶500.

### Cell Lines and Culture Conditions

COS7 cells (Lombardi Co-Resources Cancer Center, Georgetown University [31]) were maintained in Opti-MEM (Invitrogen) with 10% fetal bovine serum (Invitrogen) and 1% penicillin-streptomycin (Invitogen) in 5% CO_2_. Cells were transiently transfected with 0.67–2 µg of total plasmid DNA using FuGENE 6 (Roche) according to manufacturer’s protocol. After twenty hours, cells were collected in RIPA Lysis Buffer (Millipore; 50 mM Tris-HCl, pH 7.4, 150 mM NaCl, 0.25% deoxycholic acid, 1% Nonidet P-40, and 1 mM EDTA) or IP buffer (see below). For PP2 and cycloheximide treatments, media was changed to serum free media the day after transfections. PP2 (Calbiochem) or DMSO was added to the media (final concentration 10 µM) for 6 hours, after which cells were collected in RIPA buffer or IP buffer. Cycloheximide (Calbiochem) was added to the media (final concentration 100 µg/mL) for 0, 15, 30, 60, 120, and 360 minutes after which cells were collected in RIPA buffer.

### Primary neurons

Primary cortical neurons were isolated from embryonic day 18–19 Sprague-Dawley rats and plated at 175,000 cells/mL in Neurobasal Media (Invitrogen) supplemented with 2% B-27 (Invitrogen), 1% penicillin/streptomycin (Invitrogen), 0.25% 100X glutamine and 0.1% of 10 mM glutamate (Invitrogen). After 5 days *in vitro*, the media was changed to serum free media and the neurons were treated with 10 µM PP2. After 24 hours, the neurons were collected in RIPA buffer.

### Cell Surface Biotinylation

Cells were co-transfected with ApoER2 and either Fyn or empty vector. A six-well plate was used for each condition. After twenty hours in culture, the media was aspirated from all wells and phosphate-buffered saline (PBS) with or without Sulfo-NHS-SS-Biotin (Pierce; 0.5 mg/well) was added to 5 of the 6 wells. (To quantify total protein, one well per condition was collected in PBS as for Western blotting.) After 30 minutes, cells were treated with Quenching Solution (Pierce), washed in Tris-buffered saline, collected in Lysis Buffer (Pierce), and sonicated. Lysates were added to a column with immobilized NeutrAvidin gel (Pierce) for 1 hour at room temperature. Columns were washed four times and incubated with SDS-PAGE sample buffer containing dithiothreitol. Surface proteins were then analyzed by Western blot. Briefly, the samples were separated by SDS-PAGE on polyacrylamide gels, transferred electrophoretically to nitrocellulose, and blocked with 5% nonfat dry milk before overnight incubation with primary antibodies.

### Immunoprecipitations

Transfected COS7 cells were collected in immunoprecipitation (IP) buffer: 50 mM Tris-HCl (pH 8.0), 4.5 mM NaCl, 1% Nonidet P-40, with phosphatase inhibitors (Sigma), and protease inhibitors (Roche). Lysates were pre-cleared with sepharose beads for 30 minutes at 4°C. Protein-G or protein-A sepharose beads (GE Healthcare) were incubated with antibodies for 1 hour at 4°C. Beads were collected by centrifugation at 4000×rpm for 5 minutes. The beads bound to the antibodies were then incubated with the pre-cleared lysate overnight. Bead-protein complexes were washed 5 times with IP buffer and resuspended in SDS sample buffer containing dithiothreitol. The samples were separated by SDS-PAGE. Samples defined as “Input” were lysates not incubated with beads or antibody; the percentage refers to the amount of sample loaded onto the gel compared to the amount added to beads.

### Animals

All animal experiments were conducted in compliance with the rules and regulations of the Institutional Animal Care and Use Committee at Georgetown University under animal protocol number 12-044. *Fyn* knockout mice were obtained from Jackson Laboratory. Controls were wild-type C57BL6. For comparisons of ApoER2 levels between knockout and wild-type mice, brains from three-week-old mice were homogenized in tissue homogenization buffer containing 250 mM sucrose, 20 mM Tris base, 1 mM EDTA, 1 mM EGTA, and protease inhibitors. Samples were further extracted in RIPA buffer, as described previously [30]. For immunoprecipitation studies, brains from wild-type mouse pups (taken on postnatal days 1, 5, and 15) were homogenized in IP buffer and spun at 13,000×rpm for 10 minutes at 4°C. Supernatants were used for IP studies.

### Statistical Analyses

All data were analyzed using analysis of variance (ANOVA) with Graphpad Prism 4 software. Bonferroni’s multiple comparisons test was used for post hoc analyses and significance determined as *p*<0.05. All experiments were performed a minimum of three times.

## Results

### The presence of Fyn increases ApoER2 levels *in vivo* and *in vitro*

**Figure 1:**
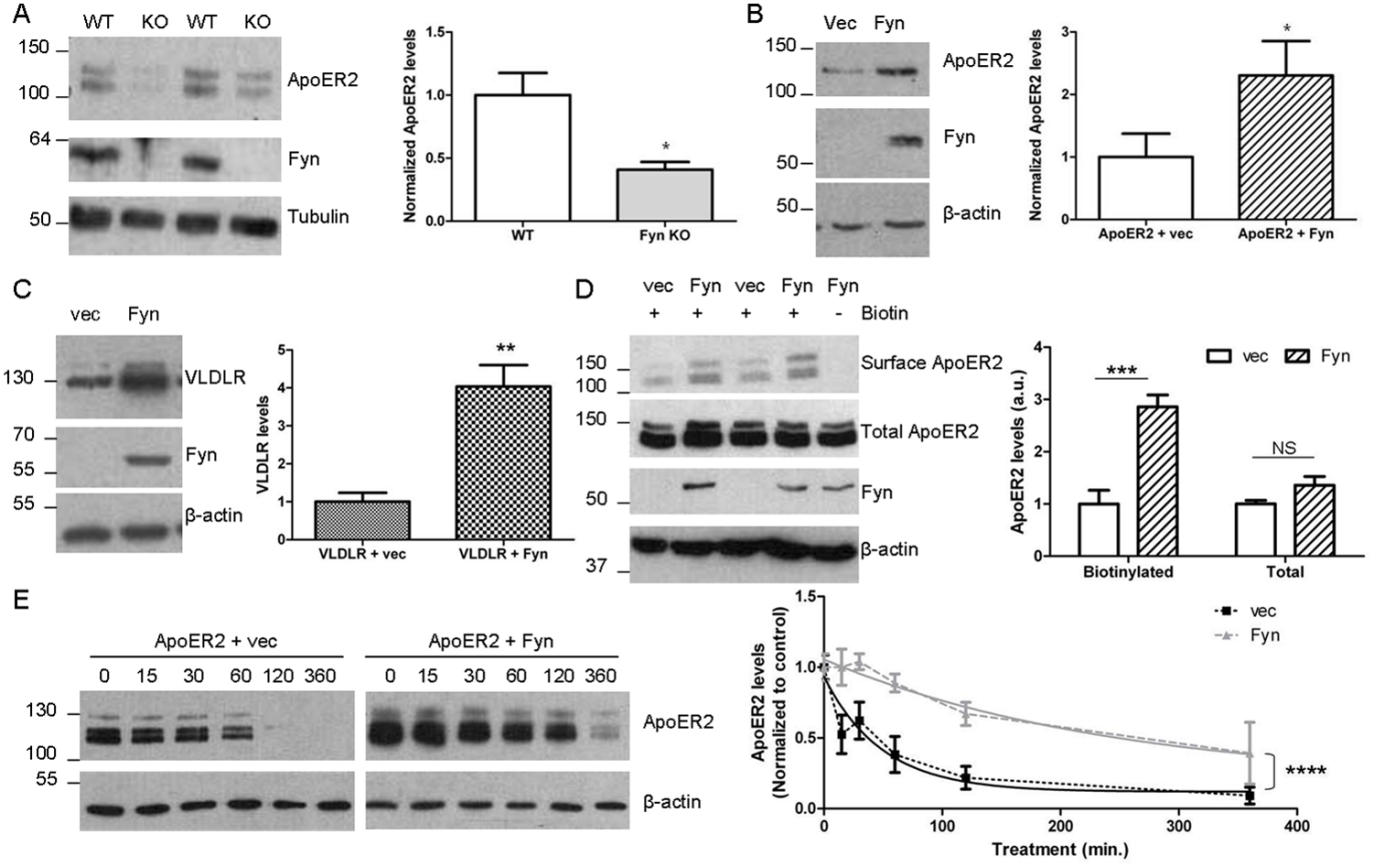
Fyn increases ApoER2 levels. A. Brains from 3 week old wild-type (WT) and *Fyn* knock-out (KO) mice were homogenized in RIPA buffer and analyzed for ApoER2, Fyn, or β-tubulin by immunoblot. ApoER2 levels were normalized to β-tubulin. *p<0.05 (t-test); Data are mean +/− SD. N = 3. B. COS7 cells co-transfected with Myc-tagged ApoER2 and empty vector (vec) or Fyn were collected in RIPA buffer and analyzed as above. ApoER2-Myc levels were normalized to β-actin. *p<0.05 (t-test); Data are mean +/− SD. N = 3. C. COS7 cells co-transfected with Myc-tagged VLDLR and empty vector or Fyn were collected in RIPA buffer, Western blotted, and probed as above. *p<0.05 (t-test). Data are mean +/− SD. N = 3. D. COS7 cells co-transfected with ApoER2 and empty vector or Fyn were subject to cell surface biotinylation. Lysates were collected, purified with an avidin gel and Western blotted for ApoER2. Corresponding cell lysates that were not biotinylated were collected in PBS and probed for total ApoER2, Fyn, or β-actin. Biotinylated (surface) and total ApoER2 levels were quantified. ***p<0.001 (Bonferroni’s Multiple Comparison test). NS: non-significant. Data are mean +/− SD. N = 3. E. COS7 cells co-transfected with ApoER2 and either empty vector or Fyn were treated with cycloheximide. Lysates were collected after 0, 15, 30, 60, 120, and 360 minutes and Western blotted for ApoER2. For graphing, levels were normalized to the 0 time point. Nonlinear regression lines (solid) are shown. ****p<0.0001; the two curves are significantly different. Data are mean +/− SD. N = 3.

We first tested whether Fyn had an effect on ApoER2 levels *in vivo*, comparing young wild-type and *Fyn* knockout mice. ApoER2 levels in brains of *Fyn* knockout animals were significantly lower by half compared to wild-type animals (Fig. 1A). Next, we examined if Fyn affected ApoER2 levels *in vitro*. COS7 cells were co-transfected with myc-tagged ApoER2 and either empty vector (as a control) or constitutively-active Fyn. In the presence of Fyn, ApoER2 levels were significantly increased by 100% (Fig. 1B).

We also examined whether Fyn increases levels of another Reelin receptor, VLDLR. In an analogous experiment to the ApoER2 experiments, we transfected COS7 cells with VLDLR and either empty vector or Fyn. In the presence of Fyn, VLDLR levels increased fourfold (Fig. 1C).

Fyn is myristoylated and membrane associated [17] and may help stabilize ApoER2 at the surface. To examine surface levels of ApoER2 in the presence of Fyn, we performed cell surface biotinylation. COS7 cells were transfected with untagged ApoER2 and either Fyn or empty vector. Sulfo-NHS-SS-Biotin was added to the media to bind to surface proteins with extracellular domains. Lysates were then collected and pulled down with an avidin gel. In the presence of Fyn, surface levels of ApoER2 were significantly increased compared to empty vector control (Fig. 1D). In this assay, untagged ApoER2 was detected with an anti-ApoER2 antibody. This antibody detects three bands of ApoER2, two smaller bands (that sometimes are indistinguishable by immunoblotting), and a larger band (Fig. 1D, bottom). The lower band was still present after cell surface biotinylation, possibly due to the overexpression of ApoER2 allowing some immature receptor to reach the cell surface. However, the banding pattern for biotinylated proteins was changed. Compared to the upper band on the total blots, the upper band on the biotinylated blots was more intense. This pattern supports the identification of the upper band as the mature, glycosylated form of ApoER2. Interestingly, total ApoER2 levels were not increased in the surface biotinylation assay (Fig. 1D). In this assay, the total ApoER2 samples were used as a control to check proper transfections and were collected in PBS, a buffer lacking detergent as opposed to the RIPA buffer used above. As discussed below, deoxycholic acid contained in RIPA buffer (but not PBS) may be better able to dissociate membranes and collect membrane-bound ApoER2.

Fyn could affect ApoER2 levels by increasing synthesis of the receptor or by stabilizing the receptor, leading to less degradation. In our *in vitro* assays, ApoER2 is driven off a CMV promoter, and we hypothesized that it was unlikely that Fyn would regulate this promoter. To test changes to ApoER2 degradation, we blocked synthesis using cycloheximide, a protein synthesis inhibitor. COS7 cells transfected with ApoER2 and vector or ApoER2 and Fyn were treated with cycloheximide for various times and ApoER2 levels were measured by Western blotting (Fig. 1E). As above, ApoER2 levels increased in the presence of Fyn. To control for this effect when comparing rates of degradation, we normalized the amount of ApoER2 present at the initial time to 1. In the presence of empty vector, ApoER2 levels steadily decreased, and we calculated the half-life of ApoER2 as 34 minutes. In the presence of Fyn, however, ApoER2 was stabilized and degraded significantly more slowly (half-life of 170 minutes) (Fig. 1E). Thus, Fyn has a demonstrable effect decreasing ApoER2 degradation and promoting ApoER2 stability.

### Fyn’s kinase activity is required for its increase of ApoER2 levels

**Figure 2:**
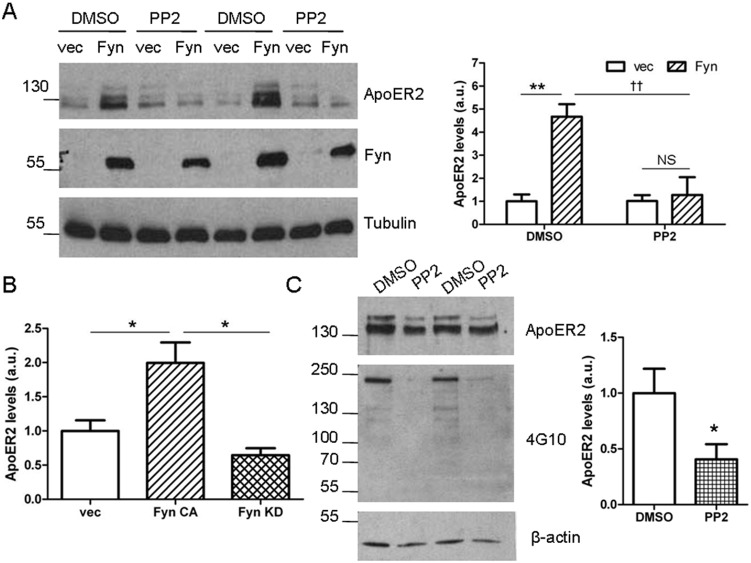
Fyn’s effects on ApoER2 levels depend on Fyn’s kinase activity. A. COS7 cells were co-transfected with ApoER2 and either Fyn or empty vector and treated with PP2 or DMSO (control) for 6 hours. Lysates were collected in RIPA buffer, Western blotted and probed for ApoER2, Fyn, and tubulin. **p<0.01 compared to empty vector treatment; †† *p<0.01 compared to control treatment with Fyn (Bonferroni’s Multiple Comparison test). NS: non-significant. Data are mean +/− SD. N = 3.* B. COS7 cells were co-transfected with ApoER2 and either empty vector, constitutively-active Fyn (Fyn CA) or kinase-dead Fyn (Fyn KD). Lysates were collected in RIPA buffer, Western blotted and ApoER2 levels were quantified. *p<0.05 (Bonferroni’s Multiple Comparison test). Data are mean +/− SD. N = 3. C. At DIV 5, primary cortical neurons were treated with PP2 for 24 hours. Lysates were collected in RIPA buffer and Western blotted for ApoER2, 4G10, and actin. Total ApoER2 levels (both upper and lower bands) were normalized to actin. *p<0.05 (t-test); Data are mean +/− SD. N = 5.

Next we asked whether the kinase activity of Fyn is responsible for its ability to increase ApoER2 levels. Cells were treated with either PP2, an inhibitor of Src family kinases, or DMSO as a control (Fig. 2A). Control cells had significantly increased levels of ApoER2 in the presence of Fyn, as above. Using the untagged construct and an ApoER2 specific antibody, we found that ApoER2 levels increased fivefold in the presence of Fyn. In PP2-treated cells, Fyn did not increase ApoER2 levels (Fig. 2A), demonstrating that Fyn’s regulation of ApoER2 levels depends on its kinase activity.

We also used a kinase-dead version of Fyn. We transfected COS7 cells with ApoER2 and either empty vector, constitutively-active Fyn (as above), or kinase-dead Fyn. All constructs were expressed in these cells. As above, ApoER2 levels were significantly increased in the presence of constitutively-active Fyn (Fig. 2B). However, in the presence of kinase-dead Fyn, ApoER2 levels were unchanged. Thus, pharmacologic and genetic approaches both indicate that Fyn kinase activity is necessary for its effects on ApoER2 levels.

To test whether the kinase activity of Fyn affects endogenous ApoER2, we treated cortical primary neurons with PP2. First, we tested the PP2 treatment was working in these cells by running a 4G10 blot for total phosphorylated proteins. Phosphorylated proteins decreased in the presence of PP2, as expected (Fig. 2C, middle blot). Next, ApoER2 levels were compared in neurons treated with DMSO and PP2, as above. In the presence of PP2, ApoER2 levels were reduced by half (Fig. 2C). The difference was more striking in the upper band, suggesting that in primary neurons, Fyn primarily affects the levels of the glycosylated form of ApoER2. These data are consistent with the effect of Fyn on decreasing ApoER2 degradation (Figure 1), although it remains possible that Fyn first affects the process of ApoER2 glycosylation, which alters its subsequent degradation. Together these data demonstrate that Fyn activity is necessary for its effects increasing ApoER2 levels.

### Increase in ApoER2 levels is independent of its intracellular domain

**Figure 3:**
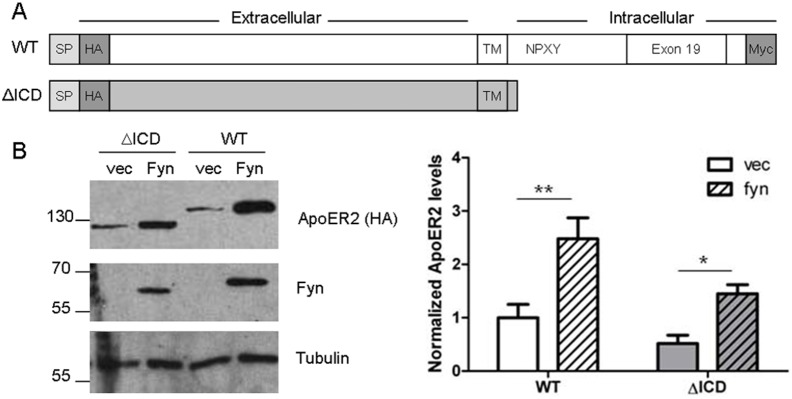
ApoER2 levels increase independently of the intracellular domain of ApoER2. A. Schematic of tagged ApoER2 constructs. Wild-type (WT) murine ApoER2 with both an N-terminal HA tag and a C-terminal myc tag. ΔICD is missing amino acids 731–841 and the myc tag. SP: Signal peptide. B. COS7 cells were co-transfected with either ApoER2 WT or ΔICD and either Fyn or empty vector. Lysates were collected in RIPA buffer and analyzed for HA, Fyn, and Tubulin. *p<0.05, **p<0.01 vs empty vector (Bonferroni’s Multiple Comparisons test). Data are mean +/− SD. N = 5.

Murine ApoER2 has three intracellular tyrosines (Y745, Y769, and Y776) that could be phosphorylated by Fyn. Y745 and Y769 are contained in exon 18, while Y776 is in the alternatively spliced exon 19. Since Fyn’s kinase activity contributes to the increase in ApoER2 levels, we hypothesized that the intracellular ApoER2 tyrosines were necessary for this effect of Fyn. We created ApoER2 constructs containing point mutations that changed the tyrosines to phenylalanines. Using a double mutant with both exon 18 tyrosines (Y745 and Y769) mutated or a construct lacking exon 19 (and Y776), we found that Fyn still increased ApoER2 levels (data not shown). Next we created a construct lacking the intracellular domain of ApoER2 (ΔICD), and thus all the intracellular tyrosines (Fig. 3A). In this ApoER2 ΔICD construct, Fyn still was able to increase ApoER2 levels (Fig. 3B). We conclude that although the kinase activity of Fyn is required for the increase in ApoER2 levels (Fig. 2), a direct, intracellular interaction between ApoER2 and Fyn is not necessary.

### Fyn phosphorylates ApoER2

**Figure 4:**
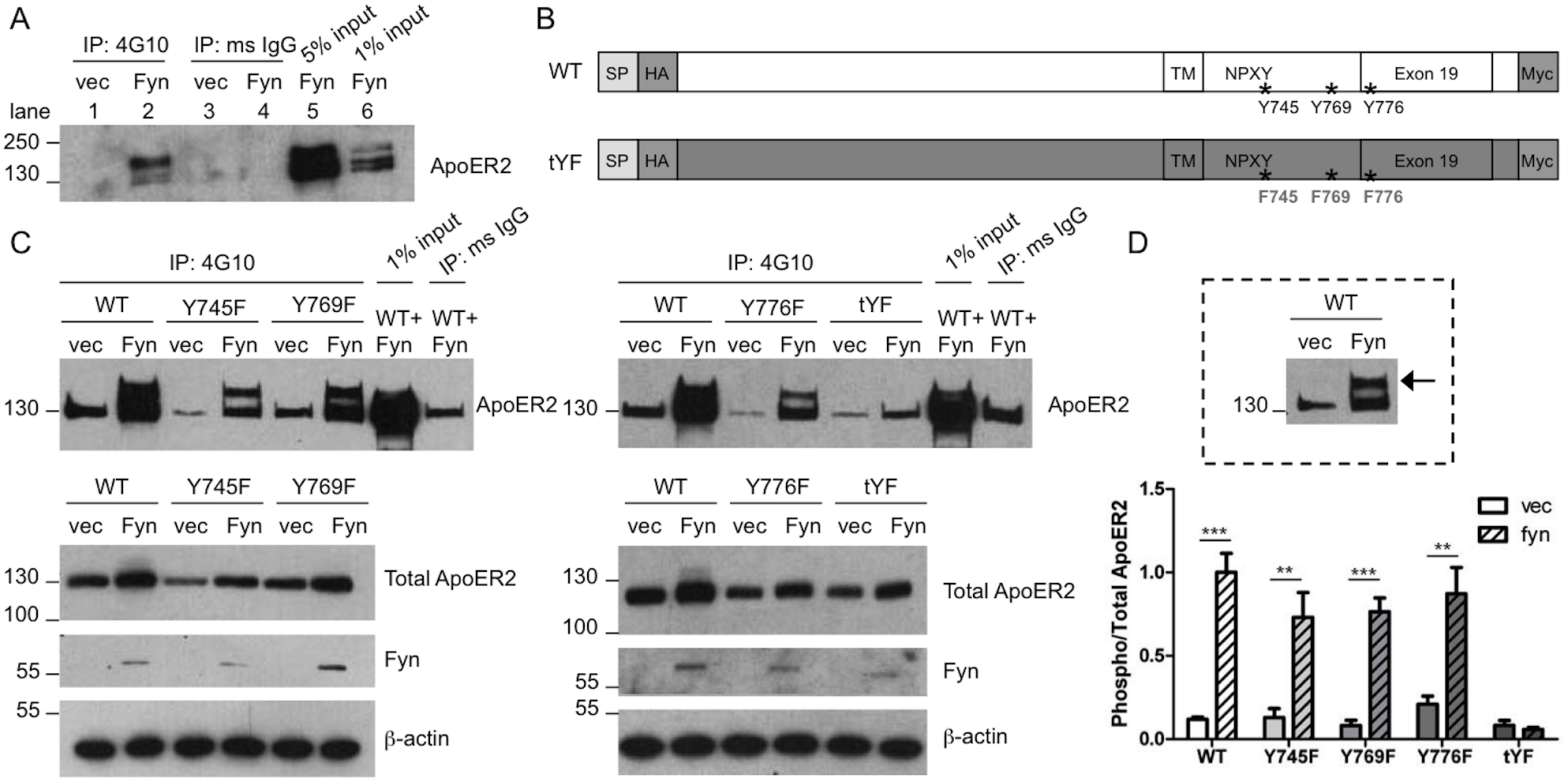
Fyn phosphorylates ApoER2. A. COS7 cells were co-transfected with untagged ApoER2 and Fyn or empty vector. Lysates were collected in IP buffer and immunoprecipitated (IP) with either 4G10 or mouse IgG, and analyzed for ApoER2. Input refers to the starting material that was not subjected to IP. Lane numbers are indicated for clarity. B. Schematic of WT ApoER2 and the mutant with all three intracellular tyrosines mutated to phenylalanines (tYF). The signal peptide (SP), N-terminal HA tag, and C-terminal myc tag are indicated. C. COS7 cells were co-transfected with different ApoER2 mutants (2 single mutants on left, 1 single mutant and triple mutant on right) and Fyn or empty vector and immunoprecipitated as in A, and analyzed for myc. Below are Western blots showing total levels of ApoER2, Fyn, and β–actin. D. Quantification of the upper-phosphorylated ApoER2 band (indicated by arrow in boxed example) over total ApoER2 from C. **p<0.01, ***p<0.001 vs empty vector (Bonferroni’s Multiple Comparisons test). Data are mean +/− SD. N = 3.

With the ApoER2 constructs we created lacking intracellular tyrosines, we were able to ask if Fyn phosphorylated ApoER2. To assay tyrosine phosphorylation of ApoER2, we transfected cells with untagged wild-type ApoER2 and empty vector or Fyn, as above. Samples were immunoprecipitated with the phosphotyrosine antibody 4G10 and probed for ApoER2. In the presence of Fyn, but not empty vector, tyrosine-phosphorylated ApoER2 was detected (Fig. 4A). By comparing the banding patterns of the immunoprecipitated lanes to the input lanes (containing a percentage of the sample that was not immunoprecipitated), we found the amount of tyrosine-phosphorylated ApoER2 in our system to be similar to the amount of ApoER2 found in 1% of total cell extract (compare Fig. 4A lane 2 to lane 6). Furthermore, the banding pattern of the tyrosine-phosphorylated ApoER2 (Fig. 4A, lane 2) was different than total ApoER2 (Fig. 4A, lane 6); phospho-ApoER2 consisted mainly of the upper band, suggesting the mature, glycosylated species is predominantly the one being phosphorylated. To ensure this phosphorylation event was due to the activity of Fyn, we treated cells with the SFK inhibitor PP2. In PP2-treated cells, tyrosine phosphorylation of ApoER2 did not occur (data not shown). Interestingly, even in the control samples, total ApoER2 levels were not consistently increased in the presence of Fyn in this assay. Here, we collected cells in IP buffer as opposed to the RIPA buffer used above (Figures 1, 2, and 3). RIPA Lysis Buffer, containing deoxycholic acid, is better able to dissociate membranes [32], releasing membrane-bound proteins such as ApoER2. IP buffer is weaker so as to maintain protein-protein intermolecular bonds. However, because the buffer is weaker, some of the ApoER2 (and other membrane-bound proteins) could remain in the membrane and be lost to our subsequent analysis. As Fyn increases surface ApoER2 (Fig. 1D), the loss of this species could explain the loss of a significant increase in total levels. These experiments were performed repeatedly with similar results.

We next investigated which tyrosine or tyrosines in ApoER2 are phosphorylated. We mutated each intracellular tyrosine to a phenylalanine. Both single mutants (Y745F, Y769F, and Y776F) and a triple mutant with all three tyrosines mutated (tYF) were created using the double-tagged ApoER2 as a template (Fig. 4B). Cells were transfected with either wild-type or mutant ApoER2 as well as either empty vector or Fyn. The next day, cell lysates were analyzed for phospho-tyrosine ApoER2. As in Fig. 4A, total wild-type and mutant ApoER2 levels were not significantly different in the presence of Fyn (due to collection in IP buffer). Furthermore, there were no differences between total levels of wild-type and mutant ApoER2.

As above, wild-type ApoER2 was phosphorylated in the presence of Fyn (Fig. 4C–D). However, with this construct and antibody, the bottom band was frequently seen, even in the control mouse IgG lanes and in empty vector conditions. Western blots for total ApoER2 showed a slightly different banding pattern compared to the untagged construct shown above. The double-tagged ApoER2 (detected with myc) had a prominent lower band and only a very minor upper band. However, when precipitated for phosphorylated ApoER2, the upper band was much more prominent, while the lower band was lessened.

Each of the single mutants (Y745F, Y769F, and Y776F) was also phosphorylated in the presence of Fyn (Fig. 4C–D), indicating that more than one of the intracellular tyrosines can be phosphorylated by Fyn. When all three tyrosines were mutated, the upper band of phospho-ApoER2 was completely lost, indicating the triple mutant (tYF) was not phosphorylated by Fyn (Fig. 4C–D).

### Dab1 promotes ApoER2 phosphorylation by Fyn

**Figure 5:**
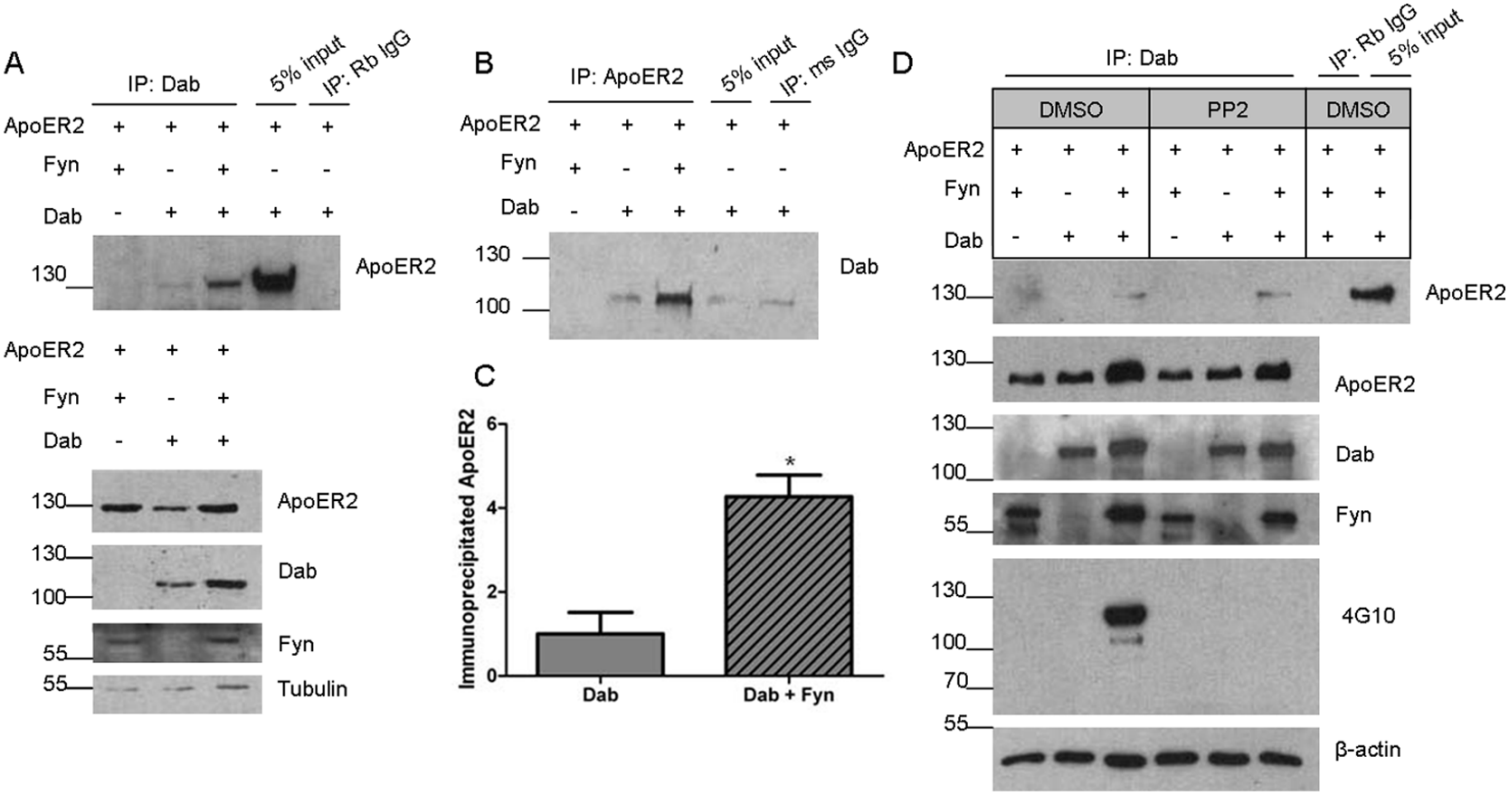
Fyn increases the interaction between ApoER2 and Dab1. COS7 cells were co-transfected with double-tagged ApoER2 and either Fyn, Dab1, or Fyn and Dab1 and lysates collected in IP buffer after 20 hours. A. Lysates were immunoprecipitated (IP) with either GFP (for Dab1) or rabbit IgG, run on a Western blot, and probed for HA (for ApoER2). Input refers to the starting material that was not subjected to IP. Below are Western blots showing total levels of ApoER2, Dab1, Fyn, and Tubulin. B. The lysates used in A were precipitated with either HA (for ApoER2) or mouse IgG, and analyzed for Dab1-GFP. C. Quantification of lanes 2 and 3 from A. *p<0.05 (t-test). Data are mean +/− SD. N = 3. D. COS7 cells were co-transfected as in A and treated with either DMSO (control) or PP2 for 6 hours. Lysates were collected and immunoprecipitated as in A.

The phosphotyrosine binding domain of Dab1 interacts with ApoER2 [12]; we investigated whether Fyn promotes this interaction. COS7 cells were transfected with ApoER2, Fyn, and Dab1 or control vector. For these experiments and those below, we used a GFP tagged Dab1 construct to ensure reliable detection (thus, GFP-Dab1 was detected around 110 kDa). After one day, cell lysates were immunoprecipitated for Dab1 and probed for ApoER2. In the presence of Fyn, the interaction between Dab1 and ApoER2 was significantly increased over threefold (Fig. 5A, C). We also performed the reverse immunoprecipitation, pulling down with ApoER2 and probing for Dab1, on the same samples and obtained similar results (Fig. 5B). We did not detect Fyn in this immunoprecipitated complex. Next, we repeated the experiment in the presence of PP2. This treatment prevented Dab1 phosphorylation, as measured by the 4G10 antibody (Fig. 5D). However, PP2 did not decrease the co-precipitation of Dab1 and ApoER2, suggesting the increased interaction between ApoER2 and Dab1 caused by Fyn is not dependent on the kinase activity of Fyn (Fig. 5D).

**Figure 6:**
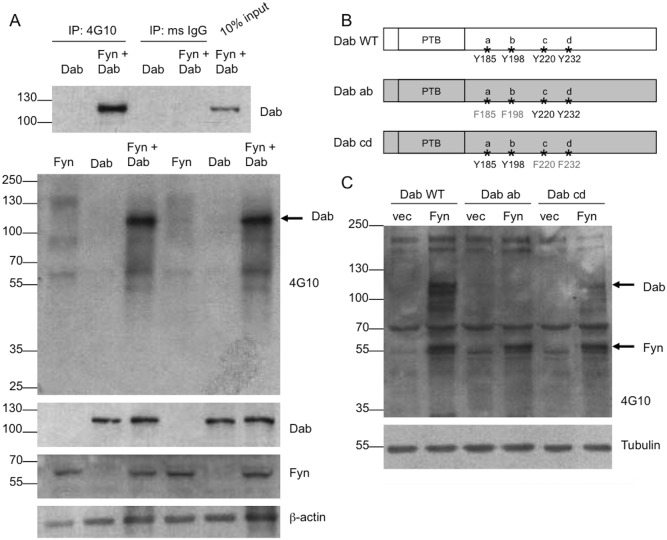
Fyn phosphorylates Dab1 first at the ab site. A. COS7 cells were transfected with Fyn, Dab1-WT, or Fyn and Dab1-WT together. Lysates were immunoprecipitated (IP, top) with either 4G10 or mouse IgG and probed for Dab1. Input refers to the starting material that was not subjected to IP. Western blots (bottom) show total levels of phosphorylated proteins (4G10), Dab1, Fyn, and β–actin. The Dab1 band is indicated (arrow). B. Schematic of Dab1 constructs. WT: Wild-type, PTB: phosphotyrosine binding domain. C. COS7 cells were transfected with Dab1-WT, Dab1-ab, or Dab1-cd and either empty vector or Fyn. Blots were probed for phosphorylated proteins, and the presumed Dab1 and Fyn bands are indicated (arrows).

In the presence of Reelin, Dab1 is phosphorylated by Fyn [6]–[7]. To detect phosphorylated Dab1 in our *in vitro* system, we transfected COS7 cells with Dab1 and either empty vector or Fyn (Fig. 6A). Again, we immunoprecipitated for phosphotyrosines (as above) and probed for Dab1. In the presence of Fyn, tyrosine-phosphorylated Dab1 constituted more than ten percent of the total Dab1 in the cell (Fig. 6A, top). Phospho-Dab1 was present at high enough levels that it could be detected on a Western blot probed for 4G10 (Fig. 6A, bottom).

Dab1 is phosphorylated by Fyn on four tyrosines, referred to as a, b, c, and d [33] (Fig. 6B). The a and b sites are phosphorylated first; if these sites are both mutated, phosphorylation at the c and d sites is also blocked [33]. We confirmed this finding in our system by examining the effect of Fyn on double mutants of Dab1 with either both the a and b sites mutated (Dab1-ab) or both the c and d sites mutated (Dab1-cd) (Fig. 6B). We confirmed similar expression of the Dab1 mutants in our system (data not shown), as previously reported [34]. Phosphorylation of Dab1-ab by Fyn was undetectable on a phosphotyrosine blot. However, Dab1-cd was phosphorylated, although to a lesser extent than wild-type Dab1 due to the loss of half of its phosphorylation sites (Fig. 6C). These experiments were done four times with similar results and these findings parallel published reports [33] showing Dab-cd mutant mice have half as much phosphorylated Dab1 compared to wild-type animals, while Dab-ab mutant mice have negligible amounts of phosphorylated Dab1.

**Figure 7:**
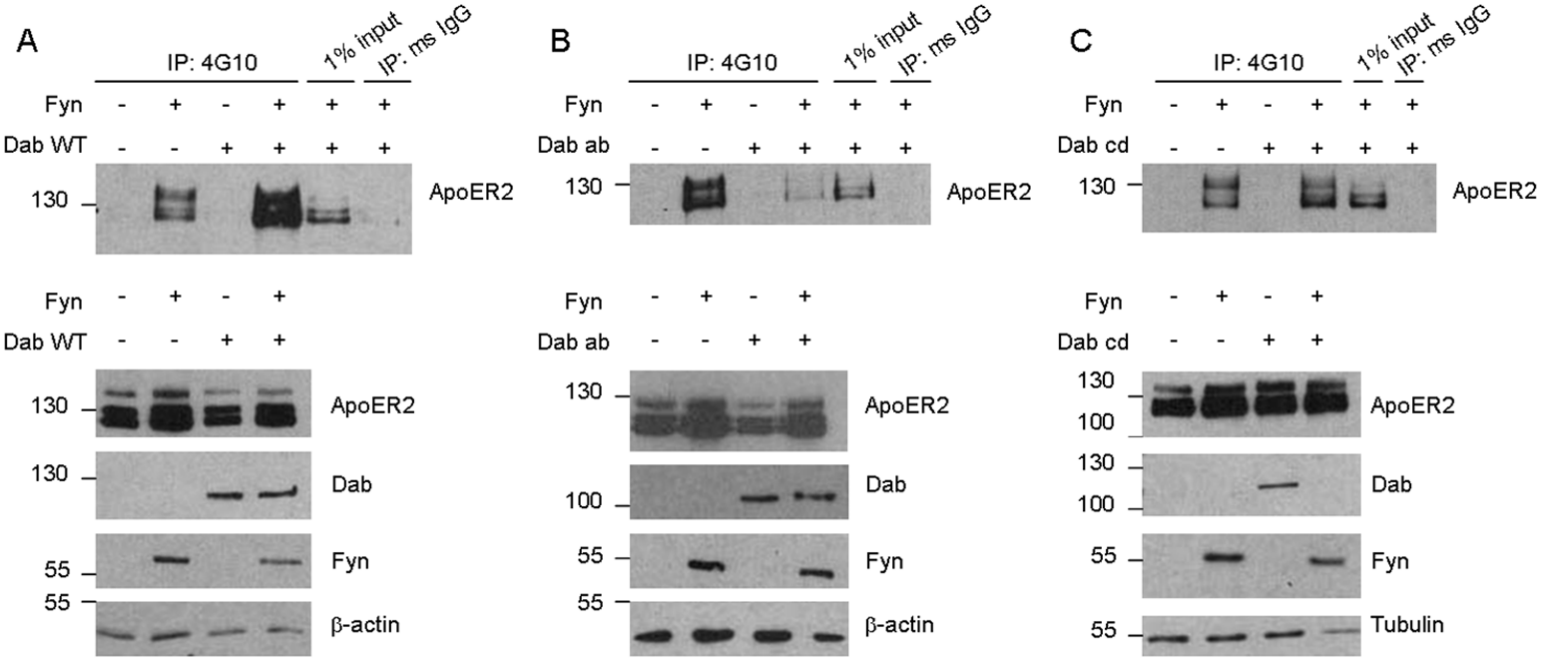
Fyn phosphorylation of ApoER2 is increased in the presence of Dab1. A. COS7 cells were transfected with untagged ApoER2 and either empty vector, Fyn, Dab1-WT, or Fyn and Dab1-WT together. Lysates were collected in IP buffer and immunoprecipitated (IP) with either 4G10 or mouse IgG, run on a Western blot, and probed for ApoER2. Input refers to the starting material that was not subjected to IP. Below are Western blots showing total levels of ApoER2, Dab1, Fyn, and β–actin. B–C. COS7 cells were transfected and analyzed as in A, but with Dab1-ab (B) or Dab1-cd (C) instead of Dab1-WT.

Since Fyn increases the interaction between ApoER2 and Dab1, and Dab1 and ApoER2 are both phosphorylated by Fyn, we asked if Dab1 plays a role in ApoER2 phosphorylation. To address this question, we transfected cells with ApoER2, Fyn, and Dab1 as above. Immunoprecipitations were performed as described above, and tyrosine-phosphorylated ApoER2 was measured. Again, phospho-ApoER2 was increased in the presence of Fyn (Fig. 7A). Interestingly, in the presence of Dab1, phospho-ApoER2 was further increased, suggesting that Dab1 promotes the phosphorylation event. In the absence of Fyn, Dab1 alone was not able to promote ApoER2 phosphorylation, consistent with Dab1 having no kinase activity. These experiments were repeated three times, with similar results.

Using the Dab1 tyrosine mutants, we next investigated if the phosphorylation of ApoER2 depended on the phosphorylation of Dab1. Cells were transfected with ApoER2, Fyn, and either Dab1-ab or Dab1-cd; tyrosine-phosphorylated ApoER2 was detected as above. In the presence of Fyn and Dab1-ab, phospho-ApoER2 was reduced compared to Fyn alone (Fig. 7B), a striking difference compared to the increase seen with wild-type Dab1 (Fig. 7A). We hypothesized that Dab1-ab could be acting as a dominant negative by binding to Fyn and preventing it from phosphorylating ApoER2. Dab1-cd did allow for phosphorylation of ApoER2 in the presence of Fyn (Fig. 7C), but phospho-ApoER2 was not increased over Fyn alone. Again, these experiments were repeated, with similar results, a minimum of three times. The difference compared to wild-type Dab1 could be due to the ability of Dab1-cd to be phosphorylated (unlike Dab1-ab) but since it cannot be phosphorylated at the ab sites, its effect on ApoER2 phosphorylation is also lessened. Interestingly, total Dab1-cd levels were lower in the presence of ApoER2 and Fyn. This effect was not observed for wild-type Dab1 or Dab1-ab. The b site has been shown to be important for Dab1 degradation [34], which would explain why Dab1-ab is not degraded compared to Dab1-cd (although wild-type Dab1 was also not degraded in our system). The difference in the effects of wild-type and mutant GFP-Dab1 indicate that the effects of Dab1 were not driven by the presence of the GFP-tag.

### ApoER2 phosphorylation *in vivo*

**Figure 8:**
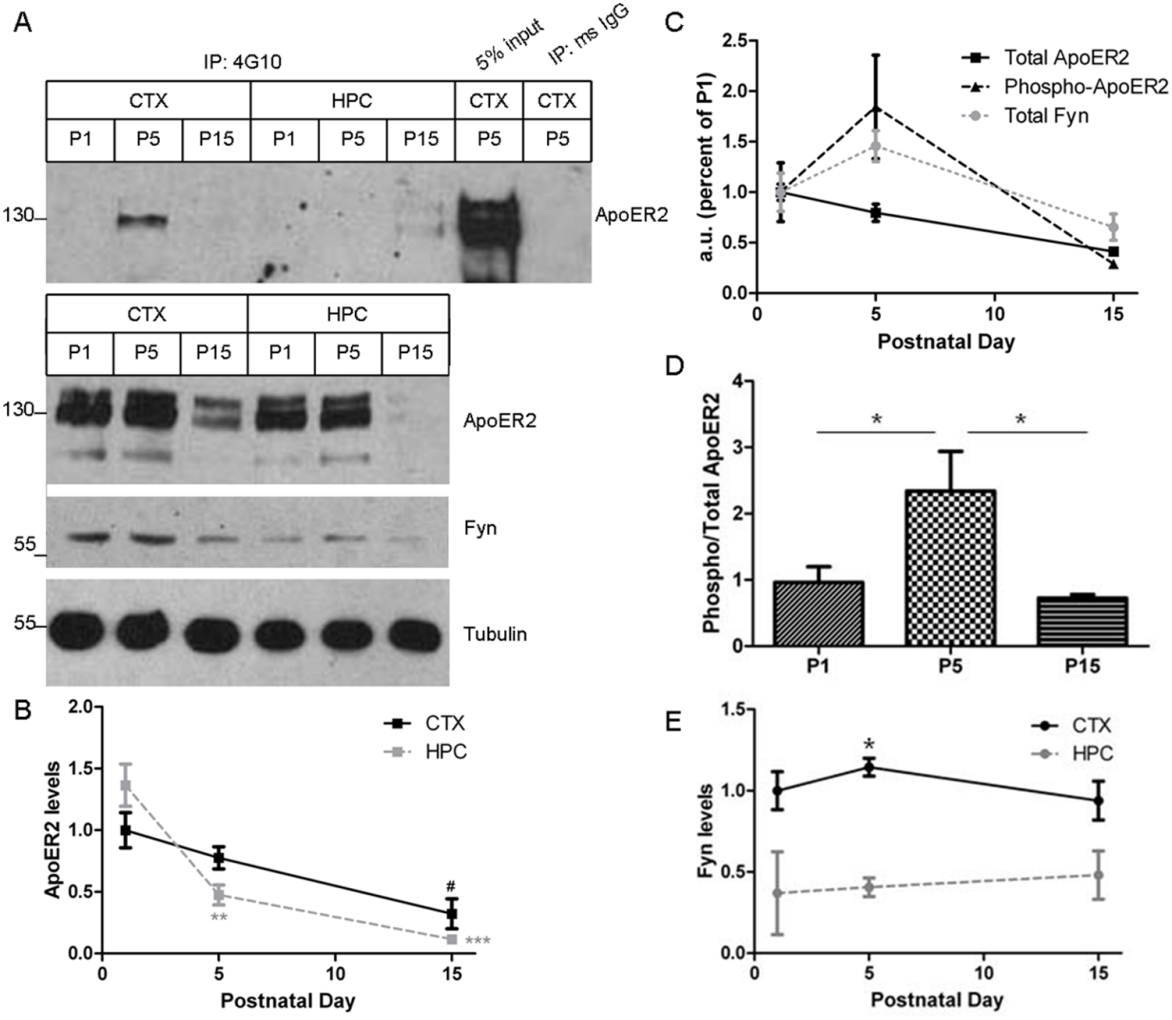
ApoER2 is phosphorylated *in vivo*. A. Cortical (CTX) and hippocampal (HPC) lysates from wild-type mice were collected on postnatal days 1, 5, and 15, homogenized in IP buffer and immunoprecipitated (IP) with either 4G10 or mouse IgG, and analyzed for ApoER2. Input refers to the starting material that was not subjected to IP. Below are Western blots showing total levels of ApoER2, Fyn, and Tubulin. B. Levels of total ApoER2 were plotted from Western blots in A. #*p<0.05 cortical P1 vs. P15;* **p<0.01 hippocampal P1 vs. P5, ***p<0.001 hippocampal P1 vs. P15. (Bonferroni’s Multiple Comparisons Test). Data are mean +/− SD. N = 3. C. For the cortical samples, levels of total ApoER2, phosphorylated ApoER2, and total Fyn were plotted. D. For the cortical samples, a ratio of phosphorylated ApoER2 to total ApoER2 was calculated. *p<0.05 (Bonferroni’s Multiple Comparisons Test). Data are mean +/− SD. N = 5. E. Levels of total Fyn were plotted from Western blots in A. *p<0.05 (Bonferroni’s Multiple Comparisons Test). Data are mean +/− SD. N = 3.

Finally, we addressed whether ApoER2 phosphorylation occurred *in vivo*. We isolated cortex and hippocampus from wild-type mouse brains at postnatal days 1, 5 and 15. In both brain regions, total ApoER2 levels decreased with age (Fig. 8A, bottom, and Fig. 8B). However, in the cortex, ApoER2 phosphorylation peaked at postnatal day 5 (Fig. 8A top, Fig. 8C–D). Interestingly, the peak of cortical ApoER2 phosphorylation at day 5 corresponded with a day 5 peak in Fyn levels (Fig. 8C). On the other hand, ApoER2 phosphorylation in the hippocampus was very low and often undetectable (Fig. 8A, top). Compared to the cortex, hippocampal levels of Fyn were about 50% lower, with significant effects at postnatal day 5 (Fig. 8E). The lack of ApoER2 phosphorylation in the hippocampus could therefore be due to less Fyn present in the hippocampus at these ages.

## Discussion

Here, we have shown that both levels of total ApoER2 and levels of phosphorylated ApoER2 increase in the presence of Fyn. Although the effect of levels of total ApoER2 depends on the kinase activity of Fyn and is blocked by the SFK inhibitor PP2, it does not depend on the direct phosphorylation of ApoER2, as levels still increase when the intracellular domain of ApoER2 (including the tyrosines that are phosphorylated by Fyn) is removed. Therefore, Fyn has two separate effects on ApoER2 via different mechanisms.

ApoER2 is enriched in caveolae [35], a subdomain of lipid rafts [36]. ApoER2’s extracellular domain is necessary and sufficient for its raft association and also leads to its slow endocytosis rate [37]. This suggests that our ApoER2 ΔICD mutant would still be localized to lipid rafts. Fyn is also found in these detergent-resistant membrane domains [38]. Fyn leads to the stabilization of ApoER2, preventing degradation, possibly within these membrane microdomains. However, we have not addressed this directly. Furthermore, VLDLR also increases in the presence of Fyn, but is not localized to lipid rafts [37]. Therefore, Fyn may affect Reelin receptors outside of lipid rafts.

Fyn’s effect on ApoER2 levels is likely not direct, but could be due in part to an as yet unknown interacting protein that is phosphorylated by Fyn and interacts with ApoER2 extracellularly. It should be noted that, when making the ApoER2 ΔICD construct, we kept the first three intracellular amino acids intact to ensure the construct would be correctly inserted into the membrane. It is possible that interactions with Fyn–a protein present at the membrane [17]–occur very close to the surface on the intracellular leaflet. It is clear, however, that the tyrosines within ApoER2 that are phosphorylated by Fyn are not required for the increase in ApoER2 levels.

Degradation of Dab1 has already been identified as one mechanism for termination of the Reelin signal [39]. Interestingly, we observed increased degradation of our Dab1-cd construct compared to wild-type Dab1. We believe Dab1-cd had its effect on promoting ApoER2 phosphorylation before being degraded. Others have shown with electroporation studies that Dab1-cd is degraded faster than Dab1-ab, but still affects downstream signaling [33]–[34].

We observed decreased degradation of ApoER2 in the presence of Fyn. Two independent pathways are responsible for ApoER2 degradation: ubiquitination by the E3 ligase IDOL (inducible degrader of Low Density Lipoprotein Receptor) and binding by PCSK9 (proprotein convertase subtilisin/kexin type 9) [40]–[41]. IDOL requires a lysine residue downstream of the NPXY domain (NPXYXK) [40], which could possibly be blocked by interactions with Fyn. However, this could not be the explanation for the ΔICD results as the crucial lysine residue was removed. PCSK9 is a secreted protein that interacts with the extracellular domain and thus could be responsible for degradation even when ApoER2’s intracellular domain is not involved. However, the role of PCSK9 in ApoER2 degradation *in vivo* is controversial [42], and there is no known link between Src Family Kinases and PCSK9.

*In vivo* we found that total ApoER2 levels in brain decline steadily with age. Others have shown that both ApoER2 and Reelin levels are highest in the early postnatal cortex and decline rapidly thereafter [43]. As ApoER2 and Reelin are required for the development of neuronal layering [1], their abundance is necessary during these early time points. The roles of the Reelin pathway and ApoER2 in the adult brain, such as regulation of LTP and dendritic spine development [2]–[3], result in a more local effect requiring subtle fine-tuning. The other known members of this signaling pathway, Dab1 and Fyn, also are expressed in the adult brain [43]–[44], suggesting that this signaling pathway remains important after the developmental period.

Fyn’s increase of ApoER2 levels could act as a positive feedback loop, promoting Reelin signaling. Although endocytosis via ApoER2 is slow, Reelin increases endocytosis [45]. Fyn could help keep ApoER2 on the surface as receptors are being internalized. Dab1 degradation could then act to turn the signal off.

As opposed to total ApoER2, which decreased consistently with aging, phosphorylated ApoER2 peaked at postnatal day 5 in the cortex, a time point that also had a peak in Fyn levels. Compared to the cortex, the hippocampus had both lower Fyn levels and undetectable levels of ApoER2 phosphorylation. Although these data are only correlative, the alignment between levels of Fyn and levels of phospho-ApoER2 in these regions at the time points examined (high ApoER2 phosphorylation when Fyn levels are high; low ApoER2 phosphorylation when Fyn levels are low) supports our *in vitro* findings that Fyn phosphorylates ApoER2. However, evidence for direct phosphorylation of ApoER2 by Fyn will depend on experiments using purified components of this system.

Src family kinases (SFKs) such as Fyn can be activated in a number of ways. In the inactive, closed conformation, the SH2 and SH3 domains of SFKs interact with and repress the catalytic domain [46]. Two phosphorylation sites are also regulatory; one at the C-terminal (Y531 in Fyn) that must be dephosphorylated to activate the kinase and one (Y420 in Fyn) that is autophosphorylated when the kinase is activated [46]–[47]. In the presence of Reelin and Dab1, the receptors may cluster, leading to Fyn phosphorylation and activation [5]–[7]. However, the exact mechanism for activation of Fyn remains unknown. Clustering of the receptors may induce clustering of Dab1 on the receptor’s tails, which promotes interactions between Dab1 and Fyn. Phosphorylated Dab1 binds to the SH2 domain of Fyn [48], possibly triggering activation of the kinase by breaking its intramolecular binding and revealing the catalytic site. The initial phosphorylation of Dab1 may depend on a small percentage of active Fyn among the cluster of receptors and Dab1 molecules in the lipid rafts.

We found that phosphorylation of ApoER2 occurs at multiple tyrosines, as phosphorylation was lost only when all three tyrosines were mutated. It may be that only two tyrosines are phosphorylation sites and future studies using double mutants could address this question. One of the tyrosines, Y745, is within the NPXY domain, a well-characterized motif that signals internationalization of extracellular ligands [11] and is the site for interactions between Reelin receptors and Dab1 [49]. It is possible that Dab1 binding to the NPXY domain could regulate endocytosis. However, ApoER2 is significantly slower at endocytosis than other members of the Low Density Lipoprotein Receptor family [50] and is therefore thought to function primarily in signal transduction. In one study, mutant ApoER2 constructs were created that prevented Dab1 binding. In an endocytosis assay, the mutant ApoER2 had lower rates of endocytosis than wild-type ApoER2 [51]. However, the rates of both ApoER2 constructs were so much lower than the related Low Density Lipoprotein Receptor that the authors dismissed the idea that endocytosis was a major function of ApoER2 [51].

Reelin increases the interaction between ApoER2 and the phosphotyrosine binding domain of Dab1 [12] and here we show that Fyn also increases the interaction between ApoER2 and Dab1 and promotes the phosphorylation of both. Interestingly, we have also shown that Fyn promotes this interaction between ApoER2 and Dab1 even when its activity is inhibited by PP2. This suggests that the binding of these molecules may occur first, before the phosphorylation events. The presence of phosphorylatable Dab1 increases Fyn’s phosphorylation of ApoER2 further, perhaps by binding ApoER2 and recruiting Fyn to the region. Dab1 binds to unphosphorylated NPXY domains [49], suggesting that phosphorylation of Y745 may disrupt Dab1 binding and act as a mechanism for signal termination.

Alternatively, while Dab1 interacts with the NPXY domain, Fyn could interact nearby at Y769. The ability of ApoER2 to interact with both molecules simultaneously and bring them together could lead to downstream signaling. Finally, the third tyrosine, Y776, is within exon 19, an alternatively spliced exon that could allow for another level of regulation. However, Y776 is not conserved in humans (NCBI Accession #004631.4) and there is no homologous site in VLDLR (NCBI Accession #013703.2), making it unlikely to be a tyrosine of critical importance for signal regulation.

In this study we focused on the effects of Fyn levels on various signaling molecules. In addition, we have found that in primary neurons Reelin treatment increases the amount of phosphorylated ApoER2 (data not shown). Together with our results that inhibition of Fyn activity blocks its effects on ApoER2 levels, these findings support the hypothesis that alterations in Fyn activation produce our observed effects.

The ultimate functional downstream effects of ApoER2 phosphorylation remain to be elucidated. ApoER2 phosphorylation could turn off Reelin signaling (possibly by leading to the disruption of Dab1 binding) or promote further downstream signaling. The phosphorylation observed here occurs after the cortical layers are established [52]. However, the members of the Reelin pathway also have a role in dendritic spine density as *ApoER2* knockout mice and *Fyn* knockout mice have reduced spine density in the cortex [53]–[54]. In addition, Reelin promotes spine density in organotypic slices, while RAP (Receptor-associated protein, an antagonist of ApoER2 and VLDLR) and PP2 decrease spine density [2]. Spines begin forming within the first postnatal week [55], the same time we detect a peak in ApoER2 phosphorylation. In addition, the Reelin pathway also has a role in dendritic growth, as Reelin promotes the development of dendritic arbors, an effect that is lost in the presence of either RAP or PP2 [56]. Therefore ApoER2 phosphorylation could have a role in dendritic spine formation or dendritic complexity. The tyrosine mutants created here can help address these questions in future studies.

Based on these findings, we propose the following mechanism for ApoER2 phosphorylation. Extracellular binding of Reelin induces clustering of ApoER2 and activates Fyn [5]–[7], [57]. Fyn promotes interaction between Dab1 and ApoER2 and phosphorylates Dab1 first on the a and b sites, followed by the c and d sites. Fyn then phosphorylates ApoER2 on any of its three intracellular tyrosines. Phosphorylated ApoER2 may disrupt binding of Dab1, allowing for Dab1’s degradation and signal termination.

In conclusion, our results clarify interactions between ApoER2 and the kinase Fyn. Through two separate mechanisms, Fyn increases both surface levels of ApoER2 and phosphorylation of ApoER2, and phosphorylation of ApoER2 is further promoted by Fyn phosphorylation of Dab1. ApoER2 phosphorylation occurs early in post-natal life, revealing a novel mechanism in Reelin signaling.
